# Mechanisms and Clinical Application of Tetramethylpyrazine (an Interesting Natural Compound Isolated from Ligusticum Wallichii): Current Status and Perspective

**DOI:** 10.1155/2016/2124638

**Published:** 2016-09-07

**Authors:** Yingke Zhao, Yue Liu, Keji Chen

**Affiliations:** ^1^Cardiovascular Diseases Centre, Xiyuan Hospital, China Academy of Chinese Medical Sciences, Beijing, China; ^2^School of Chinese Medicine, The University of Hong Kong, Pokfulam, Hong Kong

## Abstract

Tetramethylpyrazine, a natural compound from* Ligusticum wallichii* (*Chuan Xiong*), has been extensively used in China for cardiovascular and cerebrovascular diseases for about 40 years. Because of its effectiveness in multisystems, especially in cardiovascular, its pharmacological action, clinical application, and the structural modification have attracted broad attention. In this paper its mechanisms of action, the clinical status, and synthetic derivatives will be reviewed briefly.

## 1. Introduction

**Figure 1:**
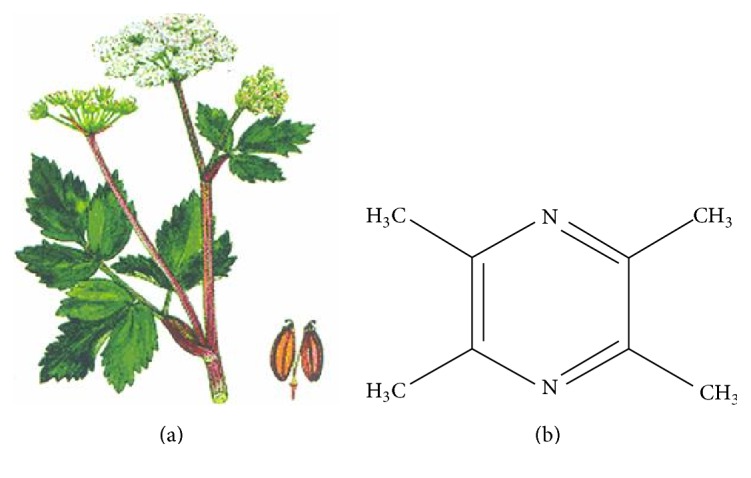
Illustration of* Ligusticum wallichii* (*Chuan Xiong*) plant (a) and chemical structure of ligustrazine (b).

Tetramethylpyrazine (ligustrazine, TMP) is a natural compound isolated from Chinese herbal medicine* Ligusticum wallichii* (*Chuan Xiong*), which has been extensively used for medicinal purpose for more than 2000 years. TMP was firstly isolated in 1957 and has been increasingly studied for its action on myocardial and cerebral infarction since 1970s [1]. In the past decades, researchers explored other pharmacological capabilities of TMP in various diseases, such as coronary heart disease, diabetes, cancers, and liver injury. Accordingly, laboratory study verified the regulation ability of this agent in multiple molecular targets, such as anti-inflammation, antioxidant, antiplatelet, and antiapoptosis. The pharmacology of TMP has been well reviewed in the past [2–4]. This paper will briefly summarize the pharmacological mechanisms and its clinical application status; moreover research about TMP derivatives, also a highly popular topic due to its inherent low bioavailability, will then be discussed [5] (see Figure 1).

## 2. Medicinal Use of TMP

### 2.1. Cardiovascular System

**Figure 2:**
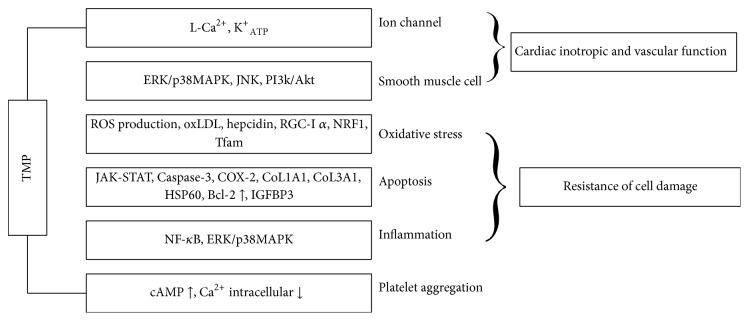
Putative mechanisms underlying cardiovascular protective effects of TMP.

The cardiovascular pharmacological effects of TMP aroused widespread interest among researchers in recent years [2, 4]. There are considerable documents supporting the view that this monomer can be a promising botanical remedy for cardiovascular diseases. The possible mechanism of its action might include modulating ion channels, stimulating the release of NO production, inhibiting vascular smooth muscle cell proliferation and migration, scavenging ROS, regulating inflammation and apoptosis, and preventing platelet aggregation (see Figure 2).

#### 2.1.1. Regulation of Cardiac Inotropic and Vascular Functions

*(1) Ion Channels*. TMP was described as “calcium antagonist” [6] and produced a vasodilation effect via inhibiting Ca^2+^ influx and the release of intracellular Ca^2+^ at first [7]. Tsai et al. [8] introduced the cultured vascular smooth muscle (A7r5) to prove that TMP can affect the calcium influx, at least partly, by mediating the opening of potassium channel. Moreover, Kim et al. [9] verified that TMP-induced vasorelaxation in isolated rat aortic rings was determined by ATP-dependent potassium channels. TMP was also reported to have a direct effect on L-type calcium current (*I*_Ca-L_), since it can reduce calcium transient in a dose-dependent manner when applied to rabbit ventricular myocyte [10]. The combination of tetramethylpyrazine phosphate (TMPP) and Ginsenoside-Rb1 (Rb1) in cTnT^R141W^ mouse model also obtained some benefit; the downregulated level of calmodulin 1 and calcium/calmodulin-dependent protein kinase II*β* (Camk2b) indicated that TMP might regulate Ca^2+^/CaM/CaMKII [11]. However, current evidence is quite preliminary; the specific link between TMP and ion channels still needs to be thoroughly investigated. Despite the potentially involved ions which had been listed in the publications, the corresponding pathways, genes, or cytokines that might be responsible for the activation of ion channels are barely understood.

*(2) Nitric Oxide Pathway*. TMP can stimulate NO production in pulmonary arteries of rat [12]; Lv et al. [13] demonstrated that Akt and the endothelial isoform of nitric oxide synthase (eNOS) phosphorylation were significantly upregulated after TMP pretreatment* in vivo*; this effect could be blocked by NO synthase (NOS) inhibitor consequently. PI3K/Akt pathway might play a pivotal role in activating eNOS and increasing NO production. Numerous researches verified the result of TMP on NO production; they believed that there are certain kinds of relationship between TMP and Akt, while the result remains controversial. Some reported TMP exerts an inhibition role in phosphorylation of Akt in N9 microglial cells [14], although others claimed that TMP can activate Akt in vascular endothelial cells [15]. Despite different cell phenotypes involved in these experiments, the relationship between TMP and Akt pathway requires further exploration. Besides, there is no clear and standard therapeutic dosages of TMP, which makes the antioxidative effect of TMP not as evident as proposed.

*(3) Smooth Muscle Cell Proliferation and Migration*. TMP can suppress the proliferation of VSMC in rabbit aortic vascular [16]. Additionally, there is a study which intended to investigate the effect of TMP on the airway smooth muscle; their data indicate that TMP might suppress the airway smooth cells proliferation via ERK1/2 signaling pathway, as the level of PDGF and p-ERK 1/2 proteins has decreased significantly in the TMP group [17]. Recently, another researcher [18] pointed out that TMP can inhibit PDGF-BB induced proliferation of VSMC, and results expressed that differentiated VSMC can be reversed by TMP and ERK and p38 MAPK might be involved in this process. Interestingly, most studies emphasized inhibitory effect on proliferation when TMP was administered before proliferation occurs, although few related mechanism or targets were identified.

#### 2.1.2. Resistance of Cell Damage

*(1) Oxidative Stress*. Clinical and laboratory studies on herbal medicine draw special attention to ROS-pathway-mediated injury in CVDs [19]. The scavenging ROS function of TMP on hypoxia induced pulmonary vascular leakage had been explored [20], and H_2_O_2_-induced human umbilical vein endothelial cells (HUVECs) were also employed to evaluate protective effect of TMP on oxidative stress, as well as its antiapoptotic properties [21]. By testing its effect on C2C12 myotube, Gao and his coworkers [22] reported that TMP could restrain mitochondrial ROS generation and upregulate the expression of PGC1, NRF1, and Tfam, which reflects mitochondrial biogenesis. TMP also exerts an endothelium protective property via downregulating the expression of ICAM-1 and HSP60 [23]. Considering the precise mechanism is still not clearly clarified, further studies focusing on the specific roles of TMP should be emphasized.

*(2) Apoptosis*. TMP can decrease the ANP mRNA expression in cardiomyocyte hypertrophy rat model and suppress the level of pJAK2, pJAK1, or pSTAT3, demonstrating that TMP can inhibit JAK-STAT signal transduction [24]. Researchers evaluated the effect of tetramethylpyrazine phosphate (TMPP) on the dilated cardiomyopathy (DCM) and reported that TMPP can prevent the progressive LV dilation and systolic dysfunction, as well as the collagen deposition and gene expression reduction of procollagens COL1A1 and COL3A1 [25]. Moreover, the upregulated level of Bcl-2 and the reduction of Caspase-3 also had been observed in apoptosis myocyte after TMP intervention [26]. TMP can exert antiapoptosis ability by inhibiting macrophage COX-2 [27]. Recently, a piece of work [28] successfully tested the protective effect of TMP in H9c2 cardiomyoblasts; the result is consistent with previous report from Zheng et al. [29].

*(3) Inflammation*. TMP can restrain LPS-induced IL-8 overexpression in HUVECs at both the protein and mRNA levels, which is possibly due to blocking the activation of the NF-kB-dependent pathway; the involvement of ERK and p38 MAPK signaling pathway has also been observed [30]. Hepcidin has emerged as a positive regulator of atherosclerotic plaque destabilization since 2007 by Sullivan [31]; its expression might be regulated by TMP [32]. SD rats with high-fat diet for 8 weeks were employed in the study; TMP group was injected with TMP at 40 mg/(kg·d), while hepcidin group was injected with heparin at 5 mg/(kg·d). After treatment, relevant markers such as blood lipid, hepcidin, ET-1, ROS, MDA, and SOD were detected. The results supported that the protective effect of TMP on endothelium might be related to inhibiting overexpressed level of hepcidin. However, direct pathways involved in regulating hepcidin need to be investigated in the future.

#### 2.1.3. Antiplatelet

Platelet aggregation plays a key role in the pathogenesis of atherothrombosis, and a variety of Chinese herbals have been examined for their antiplatelet property [33]. TMP has been commonly reported on its effect of antiplatelet since the 1980s [34]; stimulating cAMP production and inhibiting intracellular calcium mobilization were assumed to be the potential mechanism [35]. There is plenty of evidence for the suppression of platelet aggregation, although few publications were related to the platelet release reaction. A piece of research was conducted on patients who were diagnosed with acute coronary syndrome and received percutaneous coronary intervention. After TMP treatment, the level of CD63, an indicator of platelet activation, decreased significantly [36]. TMP might have an effect on inhibition of platelet release, although no experimental study is conducted to verify the role of TMP in platelet release reaction.

### 2.2. Protection on Cerebra and Spinal Cord Injury

The application of TMP in the treatment of ischemic stroke has been well documented for ages [37]. Tsai and Liang [38] directly evaluated its ability to penetrate blood brain barrier by using microdialysis technique that provides evidence for the following studies on central effect of TMP. Its neuroprotective property is partly due to modulating thioredoxin transcription [39] and downregulating the expression of neuronal isoform of NO synthase (nNOS) [40]. TMP also could attenuate the inflammation associated with ischemia by regulating the expression of NF-E2-related factor 2 (Nrf2) and heme oxygenase-1 (HO-1), which plays a role against ischemic reperfusion brain injury [40, 41]. Researchers also claimed that TMP can protect mitochondrial function and enzymatic antioxidants [42]; nevertheless sufficient evidence is needed. Recently, there is a piece of evidence for TMP on functional recovery and dendritic plasticity after ischemia [43]. The neuroprotective effects of TMP have also been tested on spinal cord injury [44, 45]. Moreover, other researchers [46] explored anti-inflammatory properties of TMP in Alzheimer's disease. Considering there is limited publications currently in this field, rigorous experiments can be warranted.

### 2.3. Cancer

Liu et al. [47] firstly investigated TMP for lymphocytes proliferation response. Later on, it has been tested on various cancers, such as leukemia [48, 49], lung cancer [50, 51], ovarian carcinoma [52], liver cancer [53], glioma [54], osteosarcoma [55], chemotherapy-resistant breast cancer [56], and prostate cancer [57]; the probable mechanism includes anti-inflammatory and promoting apoptosis. Studies intended to examine the effect of TMP derivatives like tetramethylpyrazine hydrochloride (TMPH) had yielded a similar conclusion. Large quantities of agents are currently reported as an anticarcinogen via numerous pathways, as determined in experimental environments, while few display the same effects in clinic. How to apply the laboratory finding in the clinical practice is a critical issue that remains to be settled.

### 2.4. Diabetes

As there is abundant evidence for the vascular protective action of TMP, Lee and his colleagues hypothesized to investigate this characteristic on the diabetic model at the outset. Streptozotocin-induced diabetic mice model was adopted to test lipid peroxidation level, which is one of marked pathological changes in diabetes, and the result indicated that TMP can effectively alleviate glucose and blood urea nitrogen concentration (BUN) [58]. Later on, researchers also attempt to conduct similar experiments on streptozotocin-induced diabetic nephropathy rat model; their result was consistent with the previous one on the mice model. Moreover, they demonstrated that the level of insulin, angiotensin II, and P-selectin also decreased [58, 59]. Additionally, there is a piece of study reporting protective role of TMP regarding this disorder [60].

### 2.5. Liver Injury

Liu et al. [61] detected hepatoprotective effect of TMP on acute econazole-induced liver injury. The probable explanation for this action includes inhibition of membrane lipid peroxidation [62] and oxidative stress [63]. Likewise, recent evidence indicated that TMP exerts a protective property on sepsis-induced acute liver injury mainly by ameliorating the aquaporin 8 expression [64]. Besides, there are also publications related to its inhibitory effect on hepatic fibrosis, PI3/AKT, and ERK pathways, and NLRP3 inflammasome pathway might be engaged [65, 66].

### 2.6. Renal Injury

TMP can attenuate the Cisplatin-induced nephrotoxicity in rats; antioxidative stress might be one of the proposed mechanisms [67, 68]. Similar effect had also been tested in rat renal tubular cells [69]. Its therapeutic effect on hepatic/renal ischemia-reperfusion injury in rats [70], as well as the fibrosis of renal interstitial, had been verified [71, 72]. Moreover, TMP can protect rat renal tubular cells from adriamycin-induced apoptosis [73, 74]; this action, to some extent, is due to the inhibition of p38 MAPK and FoxO1 pathways [75].

### 2.7. Others

Since TMP was reported to possess a broad spectrum of pharmacological effects, such as antioxidant, anti-inflammatory, antifibrosis effects, diseases, such as asthma and colitis, suffered from such pathological changes and had been further investigated [76–80]. However, the precise mechanism still needs to be further explored.

## 3. Current Status of Therapeutic Uses of TMP

The injection solution of TMP has been broadly used especially in China to treat ischemic stroke [81], coronary heart disease [36], diabetic nephropathy [82], and knee osteoarthritis [83]. Large amount of research is about the efficacy of TMP injection. For example, in a systematic review, they evaluated the efficacy of 22 Chinese patent medicines for stroke; there are 11 randomized controlled trials with 1652 patients related to TMP injection [84]. Although, it seems that lots of evidence have stated the efficacy, the reliability of these trials is doubtful. Few of the studies report the adverse events [82, 85, 86]; the poor methodology makes the problem even worse. Even for the treatment of the same disease, the dosage of TMP injection is quite different. Similar problem was confronted when talking about the treatment course. What is more, there are lots of combination uses of other herbal injections during the treatment, which might be difficult to figure out the interactions. Due to the low quality of clinical trials in this field, the safety concerns about the herbal injection have long been a major dispute [87].

## 4. Approaches to Improve the Bioavailability of TMP

### 4.1. Problems Associated with Drug Delivery

Early pharmacokinetic research has determined the metabolism rate of TMP and verified its* in-vivo* short half-life of *T*_1/2_ = 2.89 h. Besides, the accumulated toxicity is another potential threat to the patients for keeping an effective concentration via frequently administration [88, 89]. Considering the drug delivery deficiency of TMP [90], enormous experiments were conducted to improve its pharmacological activity since the 1990s. There are two primary approaches which can improve the biological activity.

### 4.2. Improvement of Dosage Form

The conventional TMP dosage forms include injection, oral tablet, and capsule. Conventionally, the oral administration, is the most preferable route for chronic diseases. However, this form might not be suitable, considering that hepatic first-pass metabolism can probably result in a lower bioavailability [5]. However, the intravenous injection does not help; drug concentration of injection form was peaked within 20 min but was undetected after 120 min [38]. A great deal of formulations had been proposed to solve the deficiency during drug delivery, such as porosity osmotic pump, microemulsions, ethosomes, and transdermal patch. The administration route has changed accordingly; transdermal, intranasal, intraperitoneal injection, and ocular delivery were introduced [5, 91, 92]. It is of importance to quantify the drug concentration in different sites (such as brain, liver, and skin). Research related to* in vitro* drug release,* in vivo* distribution, and the optimum dosage is urgently needed. Another unsatisfactory situation is that most of the newly designed forms were conducted at laboratory level, and extensive clinical evidence needs to be layed out to validate the clinical effect accordingly.

### 4.3. Structural Modification of TMP

**Figure 3:**
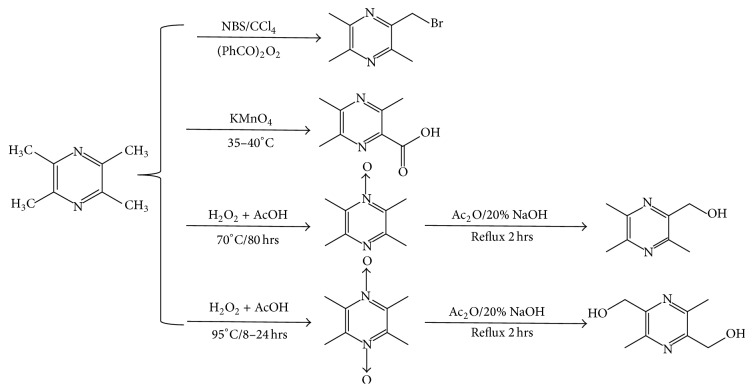
Primary intermediates of TMP.

The structural formula of TMP shows that pyrazine largely determined its pharmacodynamics, while the side chain might be mainly responsible for its pharmacokinetics. Given its inherent characteristics, the structure modification to improve the bioavailability has been broadly investigated, which opened new perspective for drug discovery. During the past decades, over 300 novel TMP derivatives had been designed and synthesized [93]. In general, most of the modifications were derived from 4 primary intermediates of TMP. They are 2-bromomethyl-3,5,6-trimethylpyrazine (TMP-Br), 3,5,6-trimethylpyrazine-2-yl (TMP-OH), 3,5,6-trimethylpyrazine-2-carboxylic acid (TMP-COOH), and 2,5-dimethylol-3,6-dimethylpyrazine (OH-TMP-OH) (Figure 3). The first step of the structural modification is the synthesis of these four fundamental compounds. With the obtained intermediates, we can easily link other reactive groups with TMP. Not only can small chemical groups be used for structural modification, but considerable herbal compounds were also introduced into synthesis process, for example, TMP-ferulic acid derivatives that combined TMP and ferulic acid, another active ingredient of* Ligusticum wallichii*, as precursors. Both TMP and ferulic acid had been extensively reported because of their antiplatelet aggregation effect [94]. Given the structural properties, calcium antagonists also could be combined with pyrazine to form new derivatives. Further study should be conducted, enabling a better understanding of the pharmacokinetics of these derivatives. After modification, further experiment is needed to demonstrate the biological activities and pharmacological characteristics of original derivatives. After 21 new derivatives had been developed, Li and his coworkers conducted experiment to evaluate the protective effect of newly designed derivatives on the HUVECs. The data proved that some of the new compounds had better protective effect compared with the reference drug [95]. Most of the results seem preferable, which reported a better effect over TMP. Although the interaction and toxicity of new derivatives have not been studied comprehensively, the active parts of the new drugs, as well as their metabolism parameters, and signal transmission might be different from TMP, which also needs to be further confirmed.

## 5. Conclusion

As an effective and multitarget product, TMP is promising and worthy of further investigation. Large amount of trials had been conducted to assess its clinical efficacy; their results seem too good to be true. So far, there is no clear evidence that gives us the standard dosage, treatment course, as well as the reporting of adverse events which is necessary for clinical trials. It would be more helpful if standardized usage for TMP was established. In that case, the results of clinical trial would be more persuasive, providing reliable evidence for decision making in practice. Despite enormous interests in the clinical uses, there is still a great deal of challenge facing the academics, such as the effectiveness, pharmacological effect, and toxicity. Nowadays, the action and mechanism of TMP have been investigated in multisystems and multidiseases, and the involved pathways and targets seem quite complex. With advances in the technique of laboratory, such as genomic, proteomic data, as well as spectrometric analysis, our understanding of herbal compounds like TMP will be more systematic. As our knowledge about its mechanism as well as disease pathophysiology has been expanded, TMP can be used more extensively and more targeted. In the future, more preclinical studies to determine its mechanisms and potential toxic effects and to answer questions related to absorption and metabolism are needed. After this preclinical work has been completed, well-designed clinical studies can be conducted. In summary, multifunction and the structural advantage make TMP a promising candidate for further study to achieve maximum therapeutic efficacy and minimum toxicity.
